# The effects of distal interlocking screws on torsional stability in three-part intertrochanteric hip fractures

**DOI:** 10.1186/s40064-015-1196-z

**Published:** 2015-08-12

**Authors:** Bryan G Vopat, Patrick M Kane, P Kaveh Mansuripur, David Paller, Sarath Koruprolu, Emily Abbood, Christopher T Born

**Affiliations:** Department of Orthopaedic Surgery, Warren Alpert Medical School of Brown University/Rhode Island Hospital, 593 Eddy Street, Providence, RI 02903 USA

**Keywords:** Intertrochanteric fractures, Cephalomedullary nails, Distal interlocks, Hip fractures

## Abstract

**Objective:**

Many surgeons currently use long cephomedullary nails for the treatment of intertrochanteric fractures. The optimal indications for deploying distal interlocks are still debatable. This study examined the torsional biomechanical properties of 3-part intertrochanteric femur fractures in a cadaveric bone model using two different distal fixation strategies, an unlocked long cephalomedullary nail versus a dynamically locked nail. Our hypothesis is that a long cephalomedullary nail does not require distal locking fixation when used for treatment of a 3-part intertrochanteric fracture.

**Methods:**

Five matched pairs of cadaveric femora were randomly assigned to one of two distal fixation treatment groups; a single distal interlock screw placed in the dynamic orientation or no distal fixation. A 3-part intertrochanteric fracture was produced. Specimens were potted and mounted in a double gimbal fixture facilitating unconstrained motion in the sagittal and coronal planes. Specimens were cyclically loaded dynamically in both internal and external rotation. Range of motion, internal and external rotation stiffness, torsion stiffness, torsion yield and ultimate torsion magnitude were calculated.

**Results:**

The samples instrumented with a distal locking screw reported statistically greater external rotational stiffness than the unlocked samples in nondestructive testing. The results of the destructive data demonstrated no statistical difference between the locked and unlocked group with regard to yield torque (p = 0.282), peak torque (p = 0.340), stiffness (p = 0.220), displacement at yield torque (p = 0.0605), and displacement at peak torque (p = 0.280).

**Conclusion:**

Distal locking of a long cephalomedullary nail increases the stiffness of the nail-femur construct in a 3-part biomechanical fracture model. However, our testing illustrates that an unlocked construct will tolerate at least equal stress before catastrophic failure in a torsional loading model.

## Background

Hip fractures are the most common fractures among the elderly in the US and are expected to further increase in frequency over the next several decades as the population ages (Cooper et al. 1992). Intertrochanteric fractures account for nearly half of all hip fractures (Forte et al. 1992). Currently, a common method of treatment of these injuries is with a long cephalomedullary nail (Anglen and Weinstein 2008). Providing a stable fracture implant construct, long cephalomedullary nails frequently allow for early mobilization, which is important in limiting morbidity in patients with these injuries (Leung et al. 1992). Cephalomedullary nails provide multiple distal fixation options with interlocking screws. However, placement of distal interlocking screws in long nails is not without drawbacks, including increased cost of hardware, longer operating times with concomitant increases in cost and anesthetic exposure, as well as increased surgical dissection and fluoroscopic radiation (Boraiah et al. 2009; Levin et al. 1987; Skjeldal and Backe 1987; Sugarman et al. 1988). Specifically, the study by Boraiah et al. found an increased operative time of 12.1 ± 3.2 min to place two distal interlocking screws using a freehand method, which corresponded to a fluoroscopy time of 28.9 ± 16.4 s (Boraiah et al. 2009). And while implant costs vary between hospitals and manufacturers, at our institution the direct cost of a sterile distal interlocking screw is roughly $85.

Given their costs, it stands to reason that the use of distal interlocking screws should be examined in order to determine which fracture patterns can be adequately stabilized without them. A recent study performed at our institution concluded that distal interlocking screws are unnecessary when treating a stable, two-part intertrochanteric fracture (Kane et al. 2013), while a study by Gallagher concluded that they should be used in unstable, four-part fractures (Gallagher et al. 2013). The aim of our current study is to determine whether distal interlocking screws significantly contribute to construct stability in three-part intertrochanteric fractures that have a stable reduction using a torsional model.

## Results and discussion

Bone density was not statistically different between locked and unlocked treatment groups (p = 0.60). The nondestructive mean external (ER) and internal (IR) rotation stiffness for intact femurs (ER: 2.58 ± 0.29, IR: 2.51 ± 0.39 Nm/°) were statistically stiffer (p < 0.05 for all) compared to fractured locked specimens (ER: 1.17 ± 0.29, IR: 0.81 ± 0.29) and fractured unlocked specimens (ER: 0.84 ± 0.16, IR: 0.57 ± 0.13). In comparing the locked specimens (ER: 1.17 ± 0.29, IR: 0.81 ± 0.29) to the unlocked specimens (ER: 0.84 ± 0.16, IR: 0.57 ± 0.13), the locked specimens had significantly greater nondestructive stiffness in external rotation (p = 0.04); there was no significant difference in internal rotation stiffness (Table 1).

**Table 1:** Fresh fracture nondestructive data

Treatment	ER stifness (Nm/°)	IR stiffness (Nm/°)	Total displacement
Locked distal fixation	1.17 ± 0.29	0.81 ± 0.29	6.47 ± 2.90
No distal fixation	0.84 ± 0.16	0.57 ± 0.13	10.89 ± 1.88
*p* value	0.040	0.409	0.998

The results of the destructive data demonstrated no statistical difference between the locked and unlocked group with regard to yield torque (p = 0.282), peak torque (p = 0.340), stiffness (p = 0.220), displacement at yield torque (p = 0.0605), and displacement at peak torque (p = 0.280). See Table 2 for a summary of these results.

**Table 2:** Destructive data for fresh fractures

Treatment	Yield torque (N M)	Yield torque (N M)	Stiffness (N M/°)	Displacement at yield (°)	Displacement at peak (°)
Locked distal fixation	6.32 ± 1.67	13.83 ± 4.77	1.35 ± 0.49	7.13 ± 2.72	24.13 ± 9.07
No distal fixation	9.38 ± 5.71	10.66 ± 5.11	0.99 ± 0.33	12.79 ± 5.24	19.14 ± 3.16
*p value*	0.282	0.340	0.220	0.605	0.280

In this model of a three-part intertrochanteric fracture, the distally locked group had increased stiffness to external rotation versus the unlocked group in nondestructive testing. However, there was no significant difference demonstrated in the destructive testing. The yield torque was greater for the unlocked specimens (9.38 Nm/°) compared to the locked specimens (6.32 Nm/°); however, this difference was not statistically significant (p = 0.282). This may indicate that by locking the nail the construct becomes too stiff for the native bone; however, given the non-significant *p* value of the yield torque, more testing is needed to investigate this hypothesis.

Similarly, the displacement to yield also approached clinical significance, and was increased in the unlocked group (unlocked: 12.79 ± 5.24; locked: 7.13 ± 2.72; p = 0.0605). This may imply that an unlocked construct can tolerate a greater degree of rotation before plastic deformity; again, however, more testing would be needed to reach statistical significance.

These findings are similar to those in our previous study testing torsional stability in locked versus unlocked treatment groups in a two-part intertrochanteric model (Kane et al. 2013), which found no differences in stiffness between the two treatment groups. Like that study, the current study suggests that in stable intertrochanteric fractures a distal interlocking screw is likely unnecessary for torsional stability. Caution must be taken when applying this study to clinical practice, as the lesser trochanter in our 3-part fracture model was held loosely in place, making it by definition a stable fracture. In order to clinically replicate the conditions of this study, the calcar region of the neck and proximal femur would have to be reduced in order to make it a stable fracture pattern. Gallagher et al. (2013) demonstrated that in four-part unstable fractures distal fixation allows significantly greater load to failure in torsion.

There are multiple techniques for placing distal interlocking screws in intramedullary nails, and while some are faster or less invasive than others, all involve marginally more cost and increased risk. Placing the distal screw involves increased OR time leading directly to increased financial cost to the patient, as well as increased anesthetic exposure (Boraiah et al. 2009). It also leads to increased fluoroscopy exposure to both the patient and OR staff, leading to increased risk of malignancy (Gugala et al. 2001; Suhm et al. 2004).

This study has several important limitations. We used fresh frozen cadaver tissue, and therefore our results will not exactly replicate in vivo biomechanical properties; however, as our construct consisted of bone and implant, with no reliance on soft tissue, we feel that these findings can be safely extrapolated to the clinical setting. In this study we chose to lock in the dynamic mode only based on the clinical preference of our senior author, however, given the shape of the dynamic distal interlocking hole, the data with respect to torsional stability would likely be very similar if the static option were utilized. Finally, a potential limitation of the study is that the fracture model used is one of a reduced calcar fragment; it merits reiteration that the findings should be extrapolated to clinical scenarios in which the calcar fragment remains nondisplaced or is reduced as part of the procedure.

## Methods

A total of 10 human femur samples (5 matched pairs, 89.3 ± 5.0 years) were used for this study. Specimens were pre-screened to exclude anatomical defects. Dual energy x-ray absorptiometry was performed on the proximal femur to acquire density data. The specimens were maintained in a freezer at −20°C until approximately 12 h prior to mechanical testing, then thawed to room temperature and skeletonized by careful dissection. All research was carried out in accordance with our institution’s ethical guidelines. As a biomechanical study, it was exempt from direct oversight by an ethics committee.

**Fig. 1:**
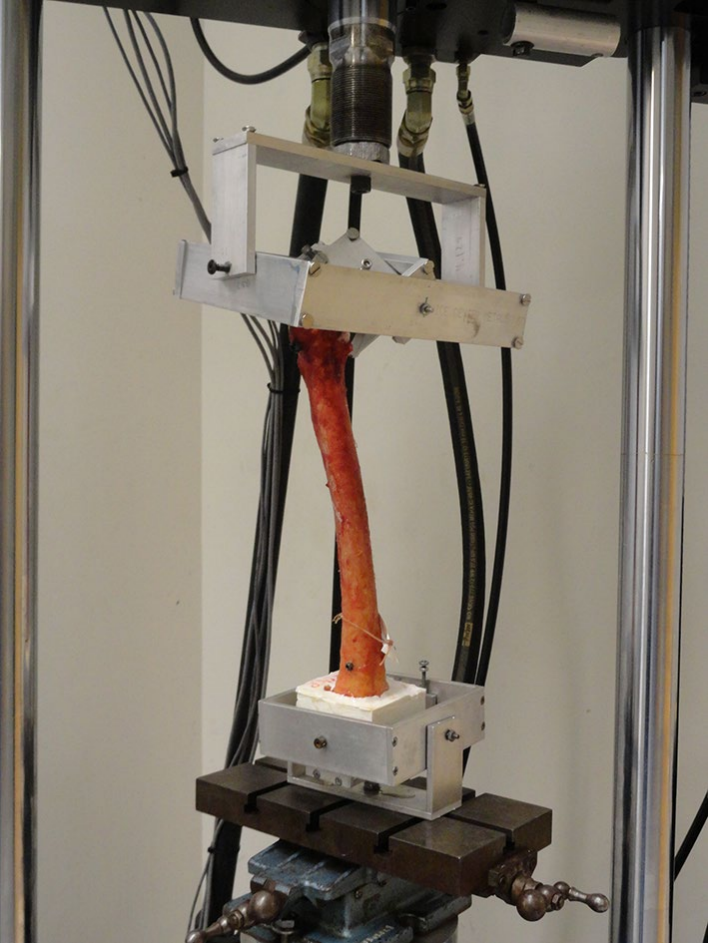
The double gimbal jig with the femur allowing unconstrained motion in the sagittal and coronal planes.

Specimens within each matched pair were randomly divided into the locked and unlocked groups. Each femur was tested without instrumentation and after the instrumented femur had an osteotomy creating a 3-part intertrochanteric fracture (Kaufer et al. 1974). The distal condyles were potted in urethane (Smooth On, Easton, PA, USA) with the assistance of a custom alignment fixture. The potted condyles were then mounted in a previously validated, double gimbal fixture facilitating unconstrained motion in the sagittal and coronal planes (Kubiak et al. 2004; Paller et al. 2012; Wahnert et al. 2011). Proximally, the head of the femur was coupled with the actuator of an Instron Biaxial Servohydraulic Load Frame (Instron Corp, Canton, MA, USA) with the use of an additional double gimbal fixture (Fig. 1). Prior to testing, samples were oriented such that the axial loading vector coincided with the center of the femoral head passing through the intercondylar notch in the coronal plane and the femoral epicondyles in the sagittal plane in the unloaded state.

Specimens were cyclically loaded for 10 cycles in both internal and external rotation to 3 Nm in load control at a frequency of 0.05 Hz for 10 cycles with a static axial compressive load of 20 N. Torque and displacement data were recorded digitally at a frequency of 25 Hz. External and internal rotation stiffness and total range of motion were quantified.

The specimens were then instrumented according to group using the Gamma3 Cephalomedullary nail system (Stryker, Mahwah, NJ, USA). A surgeon trained in the implantation of these treatment techniques performed all surgical procedures in general accordance with the Instructions for Use Guidelines. Proximally, lag screws were placed in the center–center position, as previously described by Baumgartner, for all treatments (Baumgaertner et al. 1995). Intramedullary nail and lag screw angles were measured for each femur. The proximal set-screw was placed to allow sliding of the lag screw. In the locked group, a single distal interlocking screw in the dynamic position was used. Anterior to posterior (A-P) and lateral radiographs were obtained prior to mechanical testing to insure proper implant placement and to measure tip-apex distance (TAD). All TADs were less than 25 mm.

Then, a standard three-part intertrochanteric fracture was produced by a straight sagittal saw as previously described by Rosenblum (1992). Dynamic testing of the specimens was performed. For the dynamic nondestructive test, range of motion and internal and external rotation were calculated. Following dynamic testing, samples were loaded in external rotation at a displacement rate of 10° per minute until catastrophic failure or 70° of displacement. Torsion stiffness, torsion yield and ultimate torsion magnitude were calculated during the quasi-static torque to failure test. Stiffness and torsion yield were calculated by a single blinded investigator using a custom program (National Instruments, Austin, TX, USA). In all instances, the initial linear portion of the torque versus displacement curve where the r squared value maximized was used for the calculation of stiffness. A two percent offset yield calculation was made by the same metric using a gauge length of 82.5 mm, representing a standardized distance of the distal end of the lag screw within the femoral head and its intersection with the intramedullary nail (100 mm lag screw length minus half of the mean mid-shaft bone diameter of 35 mm). Yield was defined as the intersection point of the actual torque versus displacement curve and the 2% offset line. For all specimens, the mechanism of failure was failure of the nail through the greater trochanter producing a spiral or oblique fracture pattern.

### Statistical analysis

Paired t-tests evaluated outcome variable differences for both the dynamic and torque to failure tests between treatment groups using SigmaPlot (version 12.0, Systat, San Jose, CA, USA). A paired t-test was performed to determine whether significant differences exist between the two treatment groups with regard to bone density and T-Score. In all cases, statistical significance was set to p < 0.05 a priori.

## Abbreviation

TAD: tip-apex distance
